# Identification of a CD133−CD55− population functions as a fetal common skeletal progenitor

**DOI:** 10.1038/srep38632

**Published:** 2016-12-08

**Authors:** Lihong Weng, Xingbin Hu, Bijender Kumar, Mayra Garcia, Ivan Todorov, Xiaoman Jung, Guido Marcucci, Stephen J. Forman, Ching-Cheng Chen

**Affiliations:** 1 Divison of Hematopoietic Stem Cell and Leukemia Research, Gehr Family Center for Leukemia Research, Beckman Research Institute of City of Hope, Duarte, CA 91010, USA; 2 Department of Hematology and Hematopoietic Cell Transplantation, City of Hope, Duarte, CA 91010, USA; 3 Departments of Cancer Immunotherapeutic and Immunology, Beckman Research Institute of City of Hope, Duarte, CA 91010, USA; 4 Department of Transfusion Medicine, Xijing Hospital, Fourth Military Medical University, Xi’an 7100032, P.R. China; 5 Department of Diabetes and Metabolic Diseases Research, Beckman Research Institute of City of Hope, Duarte, CA 91010, USA; 6 Irell & Manella Graduate School of Biological Sciences, City of Hope, Duarte, CA 91010, USA

## Abstract

In this study, we identified a CD105+CD90.1−CD133−CD55− (CD133−CD55−) population in the fetal skeletal element that can generate bone and bone marrow. Besides osteoblasts and chondrocytes, the CD133−CD55− common progenitors can give rise to marrow reticular stromal cells and perivascular mesenchymal progenitors suggesting they function as the fetal common skeletal progenitor. Suppression of CXCL12 and Kitl expression in CD133−CD55− common progenitors severely disrupted the BM niche formation but not bone generation. Thus, CD133−CD55− common progenitors are the main source of CXCL12 and Kitl producing cells in the developing marrow.

The hematopoietic stem cell (HSC) niche is composed of diverse cellular and molecular components that are capable of influencing HSC self-renewal and differentiation^1^,^2^,^3^,^4^,^5^,^6^. In recent years there has been an intensive interest in understanding the cellular and molecular constituents of the BM niche. Many researchers have identified the cell types that comprise the BM niche and also characterized the factors that are important in regulation of BM niche function^5^,^6^,^7^,^8^,^9^,^10^,^11^,^12^,^13^. Owen and Friedstein first propose the existence of a common progenitor or stem cell that generates a range of tissues, including various stromal cells within the BM niche, to make up the skeleton^14^. Recent studies of Chan *et al*. and Worthley *et al*. identified the stem cell for skeletal tissues and its downstream progenitors in postnatal mice^6^,^12^,^13^,^15^. However, when and if such progenitors or stem cells arise in fetal tissue remain unknown.

We have previously identified a fetal osteochondral progenitor in mouse fetal skeletal elements that forms both bone and marrow cavity in an ectopic bone assay^12^. However, its contributions to the stromal compartment within the BM niche remain undefined. In this study, we further separated fetal osteochondral progenitors and found a fetal skeletal common progenitor that can generate both bone and marrow cavity in the ectopic bone forming assay and differentiated into reticular stromal cells, alpha smooth muscle actin (αSMA)/CD140α+ perivascular cells and Sca1+ mesenchymal progenitors in ectopic bones. We also provide evidence that expression of CXCL12 or Kitl within the common progenitor is important for the generation of the BM niche.

## Results

### Identification of three new subpopulations within CD105+CD90.1− fetal osteochondral progenitor

**Figure 1:**
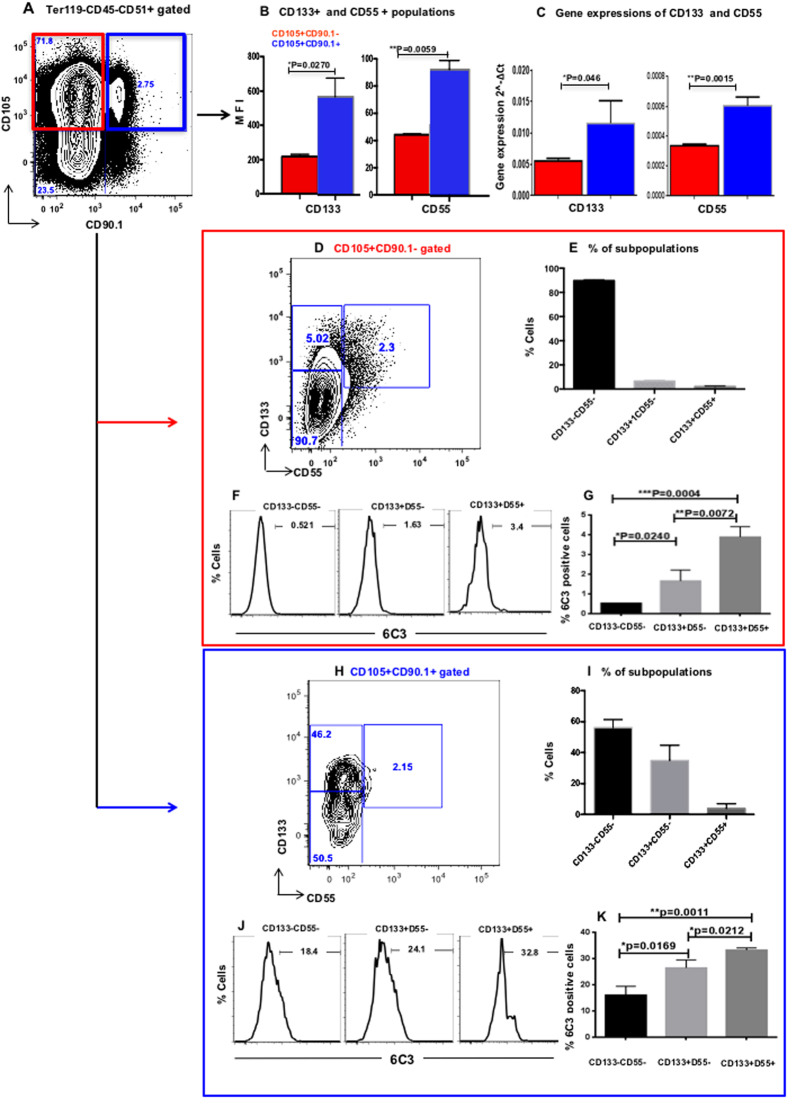
Expression of CD133 and CD55 in fetal skeletal progenitor. **(A)** Representative FACS profiles of E15.5 fetal skeletal cells. They were separated based on CD105 and CD90.1 expression into fetal osteochondral progenitors (CD105+CD90.1−) and osteoprogenitors (CD105+CD90.1+). **(B)** The expression of CD133 and CD55 in CD105+CD90.1− and CD105+CD90.1+ populations determined by FACS analysis. **(C)** The gene expression of CD133 and CD55 in CD105+CD90.1− and CD105+CD90.1+ populations. **(D,H)** Representative FACS profiles of CD133 and CD55 in CD105+CD90.1− (**D**) and CD105+CD90.1+ (**H**) populations. **(E,I)** Distribution of CD133−CD55−, CD133+CD55− and CD133+CD55+ subpopulations in CD105+CD90.1− (**E**) and CD105+CD90.1+ (**I**) populations. (**F,J**) Representative FACS analysis of 6C3 expression in CD105+CD90.1− (**F**) and CD105+CD90.1+ (**J**) derived CD133−CD55−, CD133+CD55− and CD133+CD55+ subpopulations. (**G,K**) Distribution of 6C3 positive cells in CD105+CD90.1− (**G**) and CD105+CD90.1+ (**K**) derived CD133−CD55−, CD133+CD55− and CD133+CD55+ subpopulations. (n = 3 for each group. *p < 0.05, **P < 0.010).

To identify subpopulations within the fetal osteochondral progenitor, we used CD133 and CD55 to separate the skeletal cells in E14.5 or E15.5 fetuses. CD133 is widely used as a stem cell marker in many cell types^16^,^17^,^18^,^19^,^20^,^21^,^22^, CD55 is a mesenchymal stem cell marker also used to identify endoderm in early embryonic development^23^,^24^,^25^. The cells were isolated from fetal limb skeletal elements after collagenase treatment. The non-hematopoietic CD45−Ter119− skeletal fraction was divided into CD51+CD105+CD90.1− (**CD105+CD90.1**−) and CD51+CD105+CD90.1+ (**CD105+CD90.1+**) cell populations (Fig. 1A). We then determined the expression levels of CD133 and CD55 in osteochondral progenitor (**CD105+CD90.1**−) and osteoprogenitor (**CD105+CD90.1+**) fractions by FACS. Interestingly, the expression levels of CD133 and CD55 were significantly higher in the osteoprogenitors CD105+CD90.1+ fractions (P < 0.05) (Fig. 1B, Supplementary Fig. S1). qRT-PCR analysis revealed that the gene expression levels of CD133 and CD55 were correlated with protein expressions (P < 0.05, Fig. 1C).

We next separated the osteochondral progenitor CD105+CD90.1− and osteoprogenitor CD105+CD90.1+ fractions based on the expression of CD133 and CD55. Three subpopulations, CD105+CD90.1−CD133−CD55− (**CD133**−**CD55**−), CD105+CD90.1−CD133+CD55− (**CD133+CD55**−) and CD105+CD90.1−CD133+CD55+ (**CD133+CD55+**), could be clearly distinguished within the osteochondral progenitor fraction (Fig. 1D). The CD133−CD55− was the main population (90.1 ± 0.98%) compared to the other two populations, CD133+CD55− (4.04 ± 0.80%) and CD133+CD55+ (0.59 ± 0.048%) (Fig. 1E). However, the CD105+CD90.1+ osteoprogenitor population from limb showed a much different expression pattern, the percentages of CD133+CD55− and CD133+CD55+ subpopulations increased dramatically, representing 17.7 ± 4.26% and 2.47 ± 1.40% of the total cells, respectively (Fig. 1H,I).

Previously, 6C3 was identified as a marker of osteoprogenitors in postnatal limb bone^6^,^13^. FACS analysis of E14.5 limb skeletal cells revealed that within the osteochondral progenitor fraction less than 1% of CD133−CD55− cells are 6C3+ (0.52 ± 0.003%; Fig. 1F,G). The percentage of 6C3+ cells are increased in the other two CD133+CD55− (1.65 ± 0.32%) and CD133+CD55+ (3.88 ± 0.3%) subpopulations (Fig. 1F,G). Expressions of 6C3 were much higher in the subsets from CD105+CD90.1+ osteoprogenitor fraction (16–33.2%; Fig. 1J,K). These results indicated that 6C3+ cells are relatively rare in E14.5 fetal skeletal elements.

Leptin receptor (LEPR)-expressing^1^,^26^ and Nestin-expressing^9^ perivascular mesenchymal stromal cells were reported to be major components of the adult BM niche. FACS analysis revealed that LEPR expression is rare in E14.5 fetal limbs. More cells in CD105+CD90.1+ osteoprogenitor fraction express LEPR compared to CD105+CD90.1− osteochondral progenitor fraction (0.30 ± 0.08% vs 0.06 ± 0.01%, p < 0.05; Supplementary Fig. S2A,B). Within the osteochondral progenitor, LEPR expression is the highest in the CD133+CD55− subpopulations (Supplementary Fig. S2C,D). Since there is no reliable Nestin antibody for FACS analysis, we performed qRT-PCR and found no Nestin expression in the three CD133, CD55 defined subpopulations of fetal osteochondral progenitors (data not shown). These results suggested the expression of LEPR and Nestin in skeletal progenitors was tightly regulated developmentally.

### CD133 and CD55 expression defined committed osteoprogenitors

**Figure 2:**
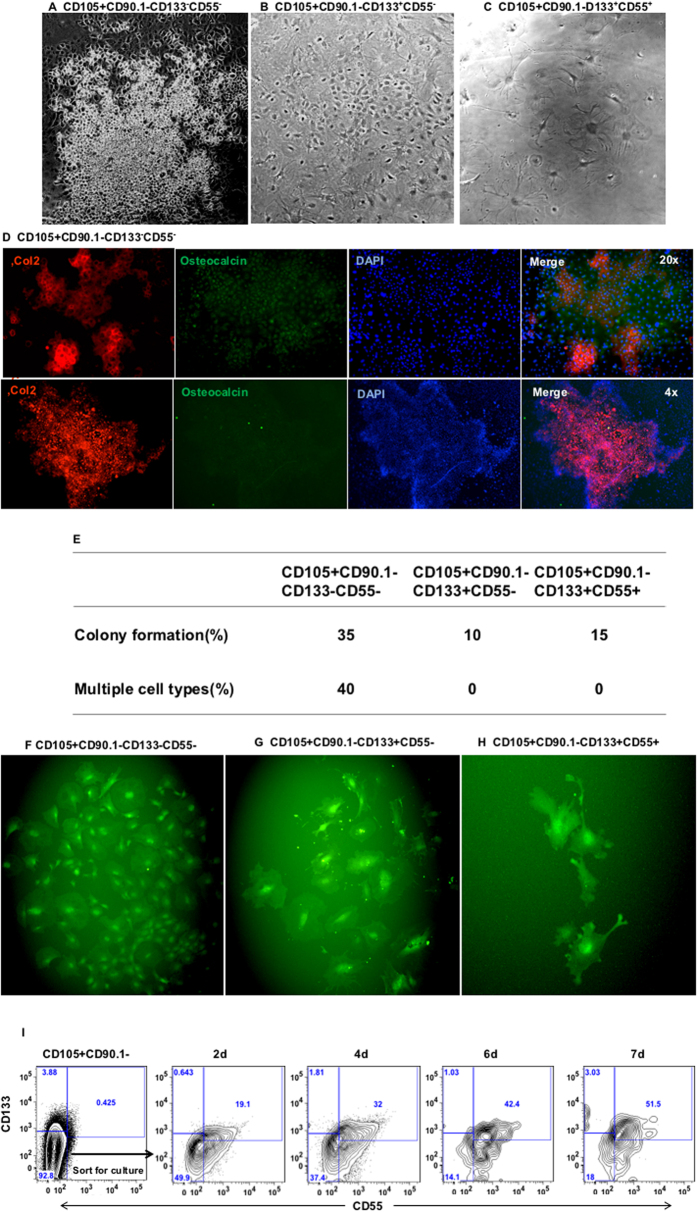
Only CD105+CD90.1−CD133−CD55− fetal progenitors generated both osteoblast and chondrocyte i*n vitro*. Fetal CD105+CD90.1− osteochondral progenitors were further sorted based on CD133 and CD55 expression and cultured for one month. **(A–C)** Bright-field image of cultured CD133−CD55− (**A**), CD133+CD55− (**B**) and CD133+CD55+ (**C**) progenitors. Only CD133−CD55− cell formed both the chondrocytes cluster and osteoblast. **(D)** Upper panels: The cultured CD133−CD55− cells were stained for the expression of Col2 (red) and osteocalcin (green). Lower panels: A representative chondrocyte cluster (red). **(E)** Summary of single cell colony forming assay of the three fetal skeletal progenitors. **(F–H)** Representative images of the colonies derived from cultured single cell of CD133−CD55− (**F**), CD133+CD55− (**G**) and CD133+CD55+ (**H**) progenitors. **(I)** Representative FACS profiles of CD133−CD55− cells 2, 4, 6 and 7 days in culture.

To investigate the differential potential of the three subsets of fetal osteochondral (CD105+CD90.1−) progenitor *in vitro,* E14.5 or E15.5 fetal osteochondral progenitor was sorted into three subpopulations, CD133−CD55−, CD133+CD55− and CD133+CD55+. The sorted cells were cultured in MEM-alpha medium for a month. The CD133−CD55− cells grew faster than the other two cell populations. Only CD133−CD55− cells were able to form chondrocyte colonies (small round cell cluster, Fig. 2A), the other two populations showed osteoblast morphology (Fig. 2B,C). Immunostaining with chondrocyte marker Col2 and osteoblast marker osteocalcin showed that the CD133−CD55− population is capable of forming both chondrocytes and osteocytes in culture. The cells within the chondrocyte cluster expressed high level of Col2 (Fig. 2D, up and low panels). We next performed a single cell culture assay to determine the colony forming capability and differentiation potential of each subpopulation. We found 35% of single cells from the CD133−CD55− population were able to form colonies after 1 month. The other two populations form colonies at a lower rate, 10% from CD133+CD55−, and 15% from CD133+CD55+ cells (Fig. 2E). 40% of the single CD133−CD55− cells that formed colonies were able to differentiate into multiple cell types with different cell morphology, whereas the other two populations showed osteoblast morphology only (Fig. 2F–H). We next investigated if the CD133−CD55− progenitor can give rise to CD133+CD55− and CD133+CD55+ subpopulations. The sorted CD133−CD55− cells were cultured in MEM-alpha medium and analyzed by flow cytometry after 2, 4, 6 and 7 days in culture. We found CD133−CD55− cells gave rise to CD133+CD55− and CD133+CD55+ subpopulations *in vitro* (Fig. 2I).

### CD133−CD55− subpopulation contributed to the bone and BM niche

**Figure 3:**
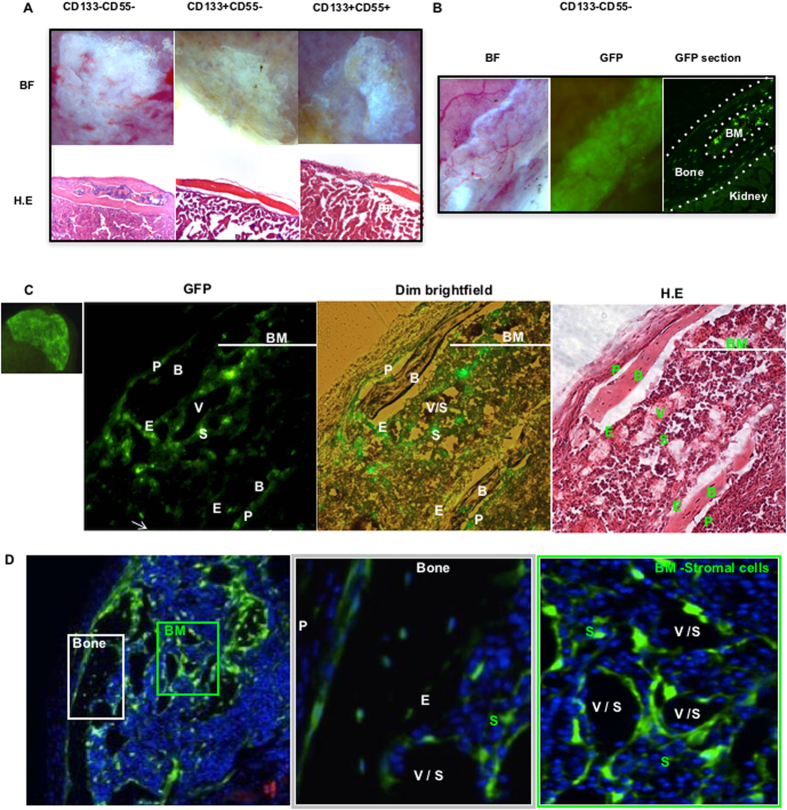
CD133−CD55− fetal progenitors contributed to ectopic bone and marrow formation *in vivo*. **(A)** Upper panel: Brightfield images of KC grafts from three sorted fetal progenitors 1 month after transplantation. Lower panel: Hematoxylin-and-Eosin (H.E) stained sections of KC grafts. **(B)** Brightfield (left) and GFP (middle) images of GFP-labeled CD133−CD55− progenitors one month after transplant. GFP image of the KC graft section (right). The KC graft was marked between the two dash lines. Marrow (BM) was located in the center. **(C)** GFP image of GFP-labeled CD133−CD55− progenitor KC graft (Far left). Images of the KC graft section in GFP(mid-left), GFP+dim light (mid-right). H.E staining of the slide after fluorescence images were captured (far right). **(D)** iCys scanning of the KC graft (left) and higher magnification of ectopic bone (middle, white square) and marrow (right, green square). P, periosteum; B, bone; V, vasculature; S, sinusoid; E, endosteum; BM, bone marrow.

We next examined the differentiation potentials of three identified subpopulations of osteochondral progenitor *in vivo*. The collected cells were mixed with matrigel and then transplanted beneath the kidney capsule (KC). After one month, the kidney grafts were harvested. We found the CD133−CD55− subpopulation was capable of forming bone and marrow cavity, the other two subpopulations formed bone only (Fig. 3A, Supplementary Table S1). Similar results were obtained in the long-term (4 month) ectopic bone forming assay (Supplementary Fig. S3). To confirm the cell origin in grafts, we sorted GFP labeled CD133−CD55− subpopulation from limb and transplanted them into KC; The GFP+ cells were found in both bone and marrow regions in grafts derived from CD133−CD55− cells (Fig. 3B). Together with *in vitro* differential assay, these results demonstrated that fetal CD133−CD55− cells are the progenitor that contributes to both bone and BM stromal cells whereas the other two subpopulations formed bone only indicating their characteristics of committed osteoprogenitors.

Furthermore, we analyzed cell components of ectopic grafts that generated from GFP labeled CD133−CD55− progenitor, 36.35% ± 0.78 of GFP+ population is CD133−CD55−, 53.75% ± 4.74 is CD133+CD55− and 1.76% ±  0.28 is CD133+CD55+, respectively (Supplementary Fig. S4). The results suggested that CD133−CD55− progenitor is capable of differentiating into CD133+CD55− and CD133+CD55+ populations during the formation of ectopic bone and BM niche.

Histological studies of the kidney graft revealed that the GFP+ cells were found in periosteum (P), bone (B), endosteum (E) and stromal (S) compartments (Fig. 3C,D). Moreover, the GFP+ cells surrounded the vasculature or sinusoids (V/S) in the marrow. The GFP+ cells connected with each other and formed a net of connective tissue to support HSCs (Fig. 3C,D). Furthermore, we did not observe any GFP+ adipocytes in CD133−CD55− generated grafts (Supplementary Table S1).

**Figure 4:**
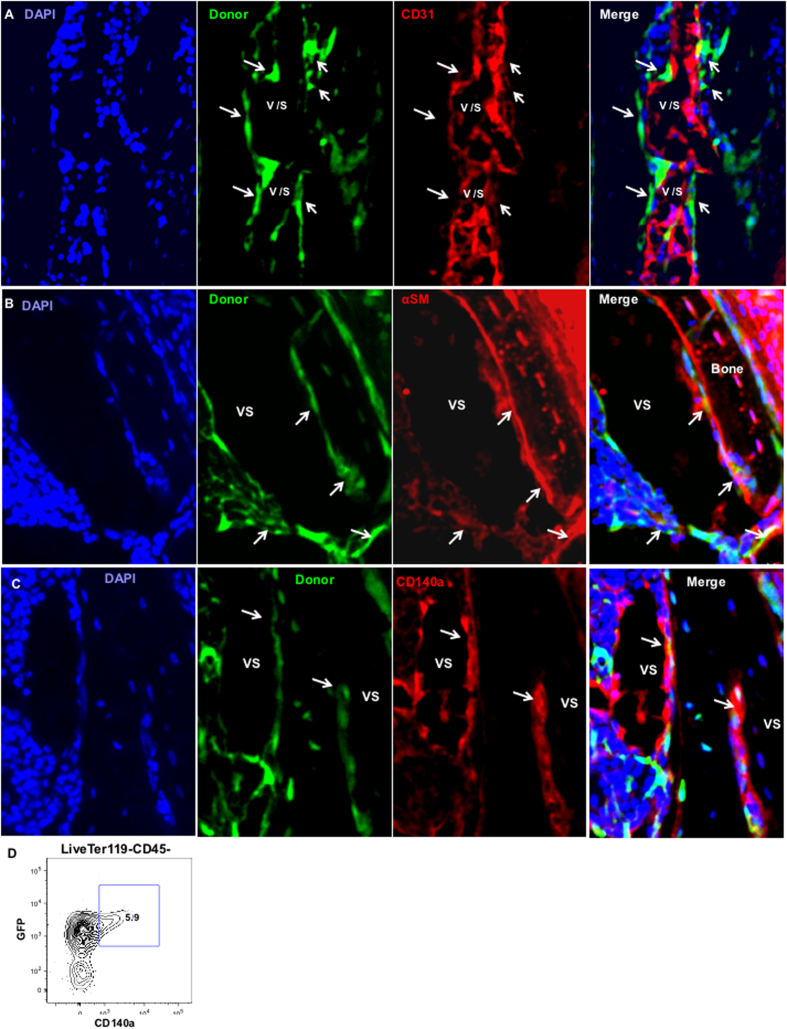
CD133−CD55− common progenitors contributed to perivascular cells but not vasculature *in vivo*. **(A–C)** iCys scanning of KC graft sections derived from GFP labeled CD133−CD55− progenitor. The sections were stained with antibodies against CD31(**A**), αSMA (**B**) or CD140a (**C**). Arrows indicate locations of donor-derived green cells. (**D**) Representative FACS analysis of CD140a expression in GFP+ graft cells.

To determine if the CD133−CD55− progenitors contributed to the vasculature in the BM niche, we identified the endothelial cells using CD31 antibody. We observed no overlap between GFP+ cells and endothelial cells (red). The GFP+ cells resided closely or were adjacent to endothelial cells and occupied the periluminal wall of the vasculature or sinusoids (Fig. 4A). We next performed immunofluorescent staining of αSMA and CD140a, the specific markers of perivascular cells^27^,^28^,^29^, on graft sections. We found overlap between GFP+ cells and the αSMA+ or CD140a+ cells (Fig. 4B,C). FACS analysis revealed that CD140a+ cells are all GFP+ (Fig. 4D). While a large portion of GFP+ cells are CD140a− as determined by FACS analysis, the portion of GFP+ cells that express CD140a may be higher. In our analysis of adult BM mesenchymal progenitor^30^, we routinely found CD140a gene expression in cells define as CD140a− by FACS analysis using single cell qPCR. Sample processing (collagenase digestion) may have weakened the CD140a staining in FACS analysis. For GFP+ cells that are not CD140a+, ~50% of them are Sca1+ cells. These results demonstrated CD133−CD55− progenitors contributed to αSMA+ or CD140a+ perivascular cells but not endothelial cells in the ectopic BM niche. Together, our results suggested CD133−CD55− cells represent fetal skeletal common progenitors.

We next analyzed the CD133 and CD55 expressions in skeletal elements during fetal development. The CD133 and CD55 expression profile of E12.5 cells was very different from other more developed fetal skeletal cells. Higher proportion of CD133+CD55− and CD133+CD55+ cells can be found in E12.5 limb cells (Supplementary Fig. S5), suggesting these cells from E12.5 limb skeletal element may be more primitive. Indeed, the E12.5 cells did not form bone or marrow cavity in ectopic bone forming assay (Supplementary Fig. S6), indicating committed skeletal progenitors did not arise until E13.5^12^. The proportion of CD133+CD55− population was briefly upregulated at E14.5 but was gradually down-regulated as the skeletal element matured. These results suggested the expression of CD133 and CD55 were tightly regulated during skeletal development.

### BM niche formation through endochondral differentiation in ectopic bone forming assay

**Figure 5:**
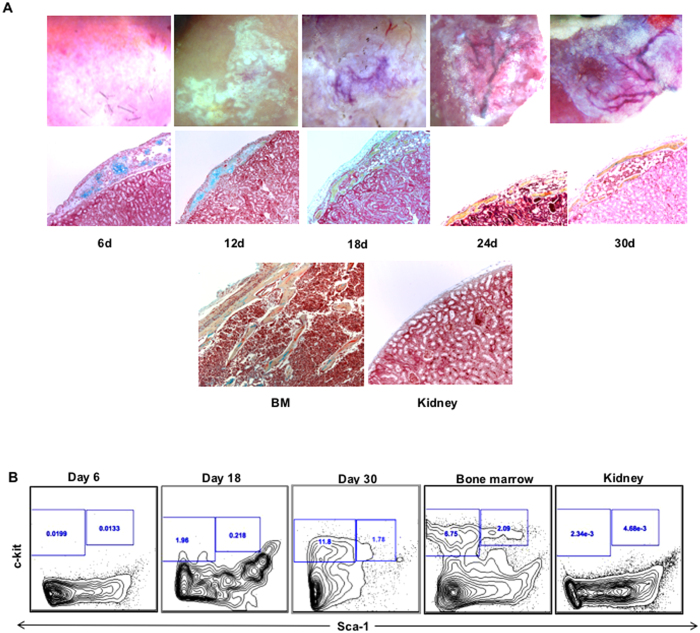
CD133−CD55− common progenitors generated ectopic BM niche through endochondral differentiation. KC grafts derived from CD133−CD55− progenitors were harvested at time indicated after transplantation. **(A)** Representative brightfield images (upper panel) and pentachrome stained sections (middle panel; yellow = osteoid, blue = cartilage) of KC grafts. The sections of adult limb bone and kidney were shown as control (lower panel). **(B)** Time course study of hematopoietic components during ectopic niche formation. Representative FACS profiles of HSC (CD45+Lineage-c-Kit+Sca1+) frequency that were pre-gated for live, CD45+lineage- cells.

We next performed time course study to monitor the ectopic bone forming process. We transplanted CD133−CD55− common progenitor under KC and the grafts were harvested on day 6, 12, 18, 24, 30 after transplantation. We found that the chondrocyte cluster occurred on day 6 and was followed by bone marrow cavity formation on day 18 (Fig. 5A middle panel). We performed pentachromal staining to detect chondrocyte clusters in E14.5 embryo, in which the bone marrow cavity has not been formed in limb and ribs yet, the chondrocytes were found in the center of limb and rib elements, but not in the mandible (Supplementary Fig. S7). FACS analysis revealed that c-kit+ hematopoietic stem and progenitor cell (HSPC) can be found in ectopic bone from day 18 and the number increased following the BM niche maturation (Fig. 5B)

### CD133−CD55−Sca1− progenitor differentiated into Sca1+ mesenchymal cells in ectopic bone

**Figure 6:**
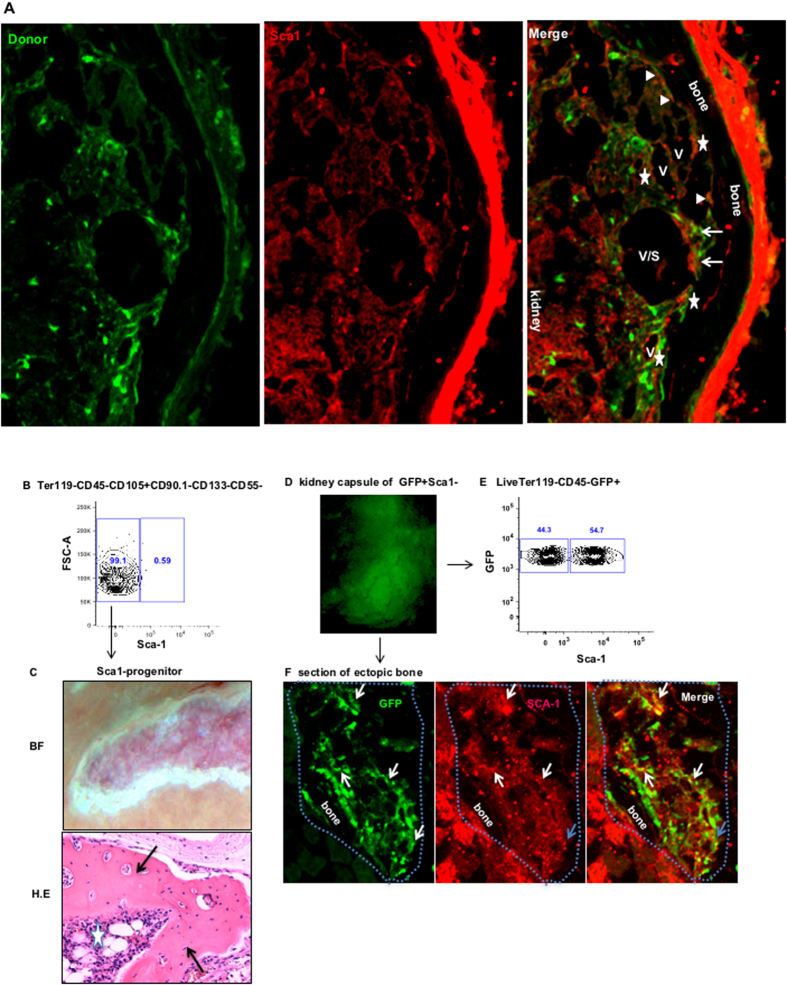
CD133−CD55− common progenitors differentiated into Sca1+ mesenchyme cells in ectopic BM niche. **(A)** Representative iCys scanned section of the KC graft derived from GFP labeled CD133−CD55− progenitor was stained with antibody against Sca1. Cells expressing both GFP and Sca1 were indicated by triangles, stars and arrows. The triangles label cells located in endosteum. The stars label the cells in perivascular regions. The arrows label the cells in stromal regions. **(B)** The expression of Sca1 in CD133−CD55− common progenitors determined by FACS analysis. **(C)** Representative brightfield (upper) and H.E section of the KC graft of sorted CD133−CD55−Sca1− cells one month after transplantation. Arrows indicate ectopic bones; star indicates the bone marrow. **(D)** GFP image of KC graft derived from GFP labeled CD133−CD55−Sca1− cells. **(E)** Representative FACS analysis of Sca−1 expression in GFP+ graft cells. **(F)** Section of GFP+ KC graft stained with Sca1. Arrows indicate the cells expressing both GFP and Sca−1. Blue dot line outlines the KC graft.

Our Lab recently identified an adult Sca1+ mesenchymal progenitor that gave rise to both osteogenic and stromal cells in the marrow^30^. The adult Sca1+ mesenchymal progenitor contributed to the stromal cells in ectopic BM niche when mixed with fetal limb cells. To determine whether the fetal CD133−CD55− common progenitor can give rise to Sca1+ mesenchymal progenitor in ectopic bone graft, we stained Sca1 in the graft generated from GFP labeled CD133−CD55− common progenitor. We found GFP+ cells overlay with Sca1+ cells in perivascular regions (Arrows), endosteal surface (Sold triangles) and stromal (Stars) (Fig. 6A). FACS analysis revealed that majority (>99%) of CD133−CD55− common progenitors are Sca1− (Fig. 6B). To determine if the Sca1+ mesenchymal progenitors in the ectopic BM niche are derived from CD133−CD55−Sca1− (**Sca1**−) cells, we sorted Sca1− cells and transplanted them under KC. After one month, Sca1− progenitor cells formed both bone (Arrows) and marrow cavity (Star) (Fig. 6C). We next analyzed kidney graft derived from GFP+ Sca1− progenitors by FACS and immunofluorescent staining. FACS analysis revealed that more than 50% of GFP+ graft cells are Sca1+ (Fig. 6E). The immunofluorescent staining showed that the GFP+ cells overlie with Sca1+ cells in the perivascular region (white arrows) and stromal (blue arrows) (Fig. 6F). These results confirmed that Sca1− progenitors gave rise to Sca1+ cells in ectopic bone.

### CXCL12 and Kitl play an important role in ectopic BM niche formation

**Figure 7:**
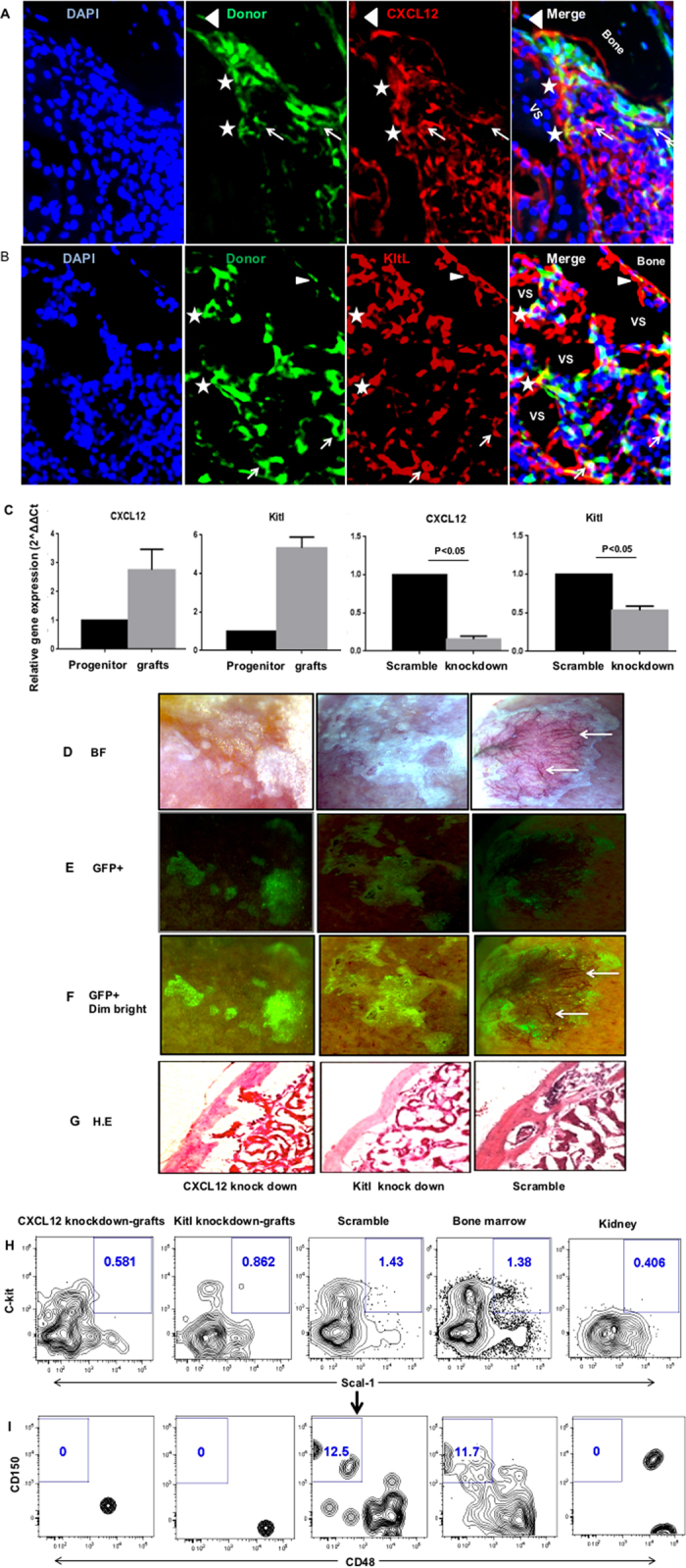
CXCL12 and Kitl play an important role in ectopic BM niche formation. **(A,B)** Representative images of sections from GFP labeled CD133−CD55− progenitors derived KC grafts stained with antibody against CXCL12 (**A**) or Kitl (**B**). Cells expressing both GFP and CXCL12 (**A**) or GFP and Kitl (**B**) were indicated by triangles, stars and arrows. The triangles label cells located in endosteum. The stars label the cells in perivascular regions. The arrows label the cells in stromal regions. **(C)** Gene expression levels of CXCL12 and Kitl in freshly sorted CD133−CD55− progenitors and GFP+ donor derived cells harvested from KC grafts. **(D)** Knockdown efficiency of CXCL12 and Kitl was determined by qRT–PCR relative to scramble control. **(E–H)** Representative brightfield (**E**), GFP (**F**), GFP + dim brightfield (**G**) and H.E section (**H**) images of KC grafts derived from common progenitors transduced with lentivirus carried CXCL12 knockdown (left), Kitl knockdown (middle) or scramble control (right) constructs. Arrows indicate the vasculatures (only presented in the scramble control). **(I,J)** Representative FACS analysis of LSK (**I**; CD45+Lineage-c-Kit+Sca1+) and LT-HSC (**J**; LSKCD150+CD48−) frequency in KC grafts that were pre-gated for live, CD45+lineage− cells. The bone marrow and kidney cells were used as positive and negative controls, respectively.

CXCL12 and Kitl are well-defined molecules that play important roles in the regulation of HSC cell fates^31^,^32^,^33^. We performed immunofluorescent staining to determine the expression of CXCL12 and Kitl in the ectopic BM niche. CXCL12 is more widely expressed in the ectopic bone (Fig. 7A). GFP+ cells expressing CXCL12 were localized at the surface of endosteal (Solid triangles), perivascular (Star) and stromal (Arrows) regions. We found that the cells in the ectopic bone expressed abundant Kitl (Fig. 7B). GFP+ cells expressing Kitl can be found at the endosteal surface (solid triangles), perivascular (Stars) and stromal (Arrows) regions. qRT-PCR analysis of GFP+ cells in grafts showed that the expression levels of CXCL12 and Kitl were significantly increased comparing to CD133−CD55− common progenitors (Fig. 7C, Left 1 and 2). The increased expression of CXCL12 and Kitl in graft cells suggested those cells might play important roles in supporting HSCs.

We next investigated if suppression of CXCL12 or Kitl expression in CD133−CD55− common progenitor will disturb ectopic BM niche formation. We knockdown the expression of CXCL12 and Kitl using GFP-labeled, lentiviral based shRNA constructs. The CXCL12 and Kitl expressions were significantly suppressed in transduced cells compared to scramble control (P < 0.05) (Fig. 7C, Right 1 and 2). The shRNA transduced GFP+ cells were sorted and transplanted into the KC. After one month, we found that down regulation of CXCL12 or Kitl severely disrupted the marrow formation but did not disturb bone formation comparing to scramble control (Fig. 7D–G). The HSC-supporting net-like structure and vasculature (arrows, Fig. 6A–C) were not observed in the ectopic bone generated by common progenitors with CXCL12 or Kitl knock-down. FACS analysis revealed that the numbers of CD45+Lineage-c-Kit+Sca1+ (LSK) cells within the ectopic bone were decreased dramatically and the LT-HSCs were not detectable in the grafts generated from the common progenitor with CXCL12 or Kitl knock-down (Fig. 7I,J). These data suggested that CXCL12 or Kitl production in common progenitor is crucial for the marrow formation but not osteoblast commitment.

## Discussion

In this study, based on CD133 and CD55 expression, we identified three subpopulations, CD133−CD55−, CD133+CD55−, CD133+CD55+, within the fetal osteochondral (CD105+CD90.1−) progenitors. While CD133+CD55− and CD133+CD55+ cells only form bone, CD133−CD55− cells are capable of forming bone and marrow cavity in ectopic bone forming assay. Besides osteoblast and chondrocyte, we found the CD133−CD55− cells gave rise to bone marrow reticular stromal cells, αSMA+/CD140a+ perivascular cells and the Sca−1+ mesenchymal progenitors in the ectopic BM niche. Thus, CD133−CD55− cells function as the common skeletal progenitor Owen and Friedstein proposed in 1988 that give rise to bone, cartilage, and hematopoiesis-supporting stroma during fetal development^34^.

While multipotent stromal progenitors have been identified in both human and postnatal mice^9^,^35^, the postnatal common skeletal stem cells were not identified until recently by two independent groups using a combination of single-cell analysis and lineage tracing technologies^6^,^15^. Chan *et al*. identified a postnatal skeletal stem cell (SSC) that can initiate skeletogenesis by producing a hierarchy of increasingly fate-limited progenitors which forming cartilage, bone and stroma in experimental models^6^. Worthley *et al*. reported a *Grem1*+ osteo-chondroreticular (OCR) stem cell that generated and maintained the articular and growth plate cartilage, bone and reticular marrow stromal cells^15^. In contrast to stromal progenitors, both SSC and OCR did not generate adipocyte *in vivo* or in *vitro*. Similarly, we did not observe significant contribution of CD133−CD55− common progenitors to adipocyte in ectopic bone forming assay, suggesting CD133−CD55− common progenitors are not the usual source of adipocytes. It fits the observation that adipogenesis in marrow is usually a later event in adult bone^36^. In contrast to OCR stem cell that did not overlap with perivascular mesenchymal progenitors, we found the fetal CD133−CD55− common progenitors give rise to adult perivascular mesenchymal progenitors in ectopic bone grafts. This discrepancy may arise from the spatial and temporal difference of these two populations in the developing and growing bones. Future studies using a lineage-tracing model are needed to delineate the relationship between fetal CD133−CD55− common progenitors and adult OCR stem cells.

Similar to previous reports^6^,^13^, we found low 6C3 expression in E14.5 fetal skeletal cells. Comparing to 6C3, CD133 and CD55 are better cell surface markers to identify committed osteoprogenitors in CD105+CD90.1− population at this developmental stage. We found more LEPR+ cells in CD105+CD90.1+ osteoprogenitor fraction suggesting LEPR-expressing cells may represent more differentiated cells in fetal limbs.

The limited expression of the adult mesenchymal stromal progenitor makers, LEPR and Nestin, in fetal limb cells suggests that there may be different waves of stem/progenitor cells contribute to development and maintenance of BM niche temporally and/or lineage-specifically^37^. However, it remains unclear if the different adult mesenchymal progenitors with proposed HSC niche functions were derived from the same multipotent stem cell. While our data indicated that CD133−CD55− common progenitors gave rise to adult Sca1+ mesenchymal progenitors, Isern *et al*. suggested that Nestin+ mesenchymal cells may have ontogenically distinct origin^38^. Additional experiments are needed to clarify if CD133−CD55− common progenitors will give rise to adult LEPR+ or Nestin+ mesenchymal progenitors.

We observed that CXCL12 and Kitl were widely expressed in CD133−CD55− common progenitor derived endosteal, perivascular and reticular stromal cells in ectopic bone. qRT-PCR showed CXCL12 and Kitl gene expression levels were dramatically increased in differentiated graft cells compared to original CD133−CD55− common progenitors suggesting that CXCL12 and Kitl expressions may be important in ectopic bone marrow maintenance. CXCL12 and Kitl have played crucial roles in maintaining HSC function, including HSC retention and mobilization in the marrow^31^,^32^,^33^. Deletion of CXCL12 from osterix–expressing stromal cell including CXCL12-abundant reticular cells and osteoblasts results in constitutive hematopoietic progenitor cell mobilization and a loss of B lymphoid progenitors^3^. Knocking down of Kitl from Leptin receptor – expressing perivascular cells depleted the HSC from marrow^1^. Unlike our previous study^12^, suppression of Kitl expression in CD133−CD55− common progenitors severely disrupted BM niche formation but bone formation in ectopic bone was not affected. This discrepancy may arise from the different populations used in these two studies. In this report, the knockdown of Kitl was performed using purified CD133−CD55− common progenitors instead of the unsorted fetal skeletal cells that contain skeletal, endothelial and hematopoietic progenitors/cells in previous report^12^. It is likely that the presence of different type of cells in the graft initially may have mitigated the effects of Kitl knockdown in skeletal progenitors by bypassing the requirements of recruiting other lineage of cells to the KC graft during the initiation of BM niche formation. More detail studies are needed to determine the actual reason of this discrepancy. Interestingly, we observed similar effects with CXCL12 knockdown study. Previous studies suggested that CXCL12/CXCR4 signaling in the mature osteoblast can feedback to regulate the osteoclast precursor pool size and play a multifunctional role in regulating bone formation and resorption^39^. Our study further indicated that CXCL12 production played critical roles in recruitment of hematopoietic cells to the niche and the initiation of BM niche formation. It also suggests the progeny of CD133−CD55− common progenitor is the main source of marrow stromal cells that are responsible for directing HSC migration through the secretion of CXCL12 to establish the BM niche in the developing marrow.

## Methods

### Mice

C57BL/Ka (B6), C57BL/Ka-CD90.1-CD45.1 (B6-CD45.1), C57BL/Ka-CD90.1-CD45.1-GFP (GFP) and B6-Rag-2^−/−^γc^−/−^ (DKO) strains were maintained in the Animal Resource Center of City of Hope under the SPF conditions. Timed embryos from B6-CD45.1 or GFP mice were used to isolate the fetal skeletal progenitors. Eight to twelve weeks old DKO mice were used as recipients for fetal skeletal cell transplantation. Mouse care and experimental procedures were performed in accordance with federal guidelines and protocols approved by the Institutional Animal Care and Use Committee at the City of Hope.

### Skeletal cell preparation, staining, isolation, KC transplantation and KC graft analysis

Fetal skeletal elements (tibia and femur) were dissected from B6-CD45.1 or GFP strain fetuses and digested in collagenase with DNase at 37 °C for 20 minutes with constant agitation. The digested cells were filtered through 40 μm filter and pelleted at 1500 rpm at 4 °C. The cell pellet was re-suspended in PBS buffer and blocked with anti-mouse CD16/32 (Cat.101302, Biolegend) antibody for 5 minutes at 4 °C. The cells were then stained with antibody cocktails of fluorochrome-conjugated antibodies against CD45 (PE/Cy5, Cat.103110, Biolegend), Ter119 (PE/Cy5, Cat.116210, Biolegend), CD51 (PE, Cat.104106, Biolegend), CD105 (PE/Cy7, Cat. 120410, Biolegend). CD90.1 (PB, Cat. 48-0900-82, eBioscience), CD133 (Percp-Cy5.5, Cat. 46-1331-82, eBioscience), and CD55 (Alexa fluor 647, Cat.131806, Biolegend) with or without Sca1 (Alexa fluor 700, Cat. 56-5981-82, eBioscience). The cells were then analyzed and sorted using FACSAria III sorter (BD), the sorted cell populations (4000–10000 cells) were mixed with 5 μl matrigel and transplanted under KC. The KC grafts were harvested, observed and imaged using a Leica M205FA stereo microscope (Leica).

To analyze the expression patterns of 6C3, Lepr and Tie2 in our newly identified populations, antibodies of 6C3 (FITC, 108305, Biolegend), Lepr (Alexa fluor 700, Cat. 56-5981-82, eBioscience) and Tie2 (APC/Cy7, self-conjugated, Cat. 124002, Biolegend) were added to above staining panel. The stained cells were analyzed by FACSAria III sorter (BD).

The KC grafts were dissociated from kidney, the graft marrow was flushed out and collected, the bone grafts were cut into small pieces and digested in collagenase with DNase at 37 °C for 30 minutes with constant rotation. The marrow or bone cells were then stained and analyzed by FACSAria III sorter (BD). The antibodies used for FACS analysis were Sca1 (Qdot605, Cat. 93-5981-42, eBioscience or Percp/Cy5.5, Cat. 122524, Biolegend), CD45 (PE, Cat. 103106, Biolegend), c-Kit (APC/Cy7, Cat. 47-1172-82, eBioscience), CD150 (PE/Cy5, Cat.115912, Biolegend), CD48 (APC, Cat, 103416, Biolegend), Lineage markers (CD3PB, CD220PB, Gr-1PB, CD11b PB, Ter119PB, Biolegend), CD140a (A700, self-conjugated, Cat. 135902, Biolegend).

Adult limb and tibia were dissected from mouse, the bone marrow was flushed out and collected as the marrow fraction and then the bone was gently crushed and digested in collagenase with DNase at 37 °C for 1 hour with constant agitation. The marrow or bone cells were processed, stained and analyzed similarly to fetal cells or KC graft cells when they were used as controls.

### Tissue section and histology

The adult femur or tibia and kidney grafts were fixed in 4% paraformaldehyde for 20 minutes at 4 °C and then decalcified in 10% EDTA buffer at 4 °C for 7 days at constant agitation. The EDTA buffer was changed every two days. The bone and kidney were then embedded in O.C.T, frozen on dry ice. The blocks were sectioned using a cryostat (Leica) equipped with the CryoJane tape-transfer system (Leica).

For Hematoxylin-and-Eosin (HE) staining, the sections were dried at RT for 20 minutes and then stained in hematoxylin for 1–3 minutes, wash in running water for 20 minutes and checked the nuclei staining under microscope, dehydrated in gradient ethanol solutions, decolorized in acid alcohol, rinsed well in tap water, counterstain in eosin, dehydrated in gradient Ethanol solutions, cleared in xylene and mounted with Permount (Fisher Chemical). For pentachrome staining, the sections were air dried at RT for 20 minutes and then stained by using Movat Pentachrome (Modified Russell-Movat) Stain Kits (StatLab) following manufacturer’s direction. The slides were observed and imaged using an Olympus IX81 inverted microscope (Olympus ).

### Cell culture

The cells from fetal skeletal elements were cultured at 37 °C in MEM alpha medium with 15% FCS under 2% O_2_. The medium was changed every 3–4 days. The cells were observed and imaged using an inverted microscope (Olympus).

### Immunofluorescence staining

The tissue sections were dried at RT for 20 minute and then blocked with 1% BSA PBS for 3×10 minutes, the sections were incubated with primary antibody at 4 °C overnight. The sections were washed with 1% BSA PBS buffer and then incubated with the secondary antibody conjugated with Alexa-dye for 1 hour at RT in dark room. The sections were washed with 1% BSA PBS buffer and stained with 0.1% DAPI for 5 minutes at RT in dark room. The sections were then washed and mounted in anti-fade mounting media (Vector Laboratories). The stained slides were observed using an Olympus IX81 microscope or scanned using an iCys Research imaging cytometer (CompuCyte).

The following antibodies were used in this study: Col2 (1:100, Cat. ab34712, Abcam), osteocalcin (1:200, Cat. Ab14173, Abcam), Kitl (1:100, Cat. sc-13126, Santa Cruz), CXCL12 (1:100, Cat. sc-6193, Santa Cruz), α-SMA (1:100, Cat. ab5694, Abcam), CD146 (1:100, Cat. 134702, Biolegend), CD140a (1:100, Cat. 135902, Biolegend), Sca-1 (1:100, Cat. 108102, Biolegend), CD31 (1:150, Cat. 5550274, BD). The secondary antibodies conjugated with Alexa fluor 488 and Alexa fluor 555 were from Invitrogen. In study with mouse primary antibody, the M.O.M. immunodetection kit (Cat. BMK-2202, Vector Laboratories) was used to eliminate the endogenous mouse immunoglobulins background in the tissue.

### Quantitative real-time PCR

RNA was extracted from sorted cells using RNeasy RNA isolation kit (Qiagen) and was reverse transcribed into cDNA using Sensiscript RT kit (Qiagen). TaqMan Gene Expression Assays (Applied Biosystems) for CXCL12 (Mm00445553_m1), Kit-ligand (Mm00442972_m1), Nestin (Mm01223404_g1) and GAPDH (Mm99999915_g1) were used to detect gene expression levels in qPCR. The CD133 and CD55 gene expression levels were detected by qPCR with primers (Supplementary Table S2) and SYBR green mastermix (Life Technologies). The PCR was performed on a ViiA 7 Real-Time PCR System (Life Technologies). The thermal cycling parameters were 40 cycles of 95 °C for 15 seconds and 60 °C for 1 minute. Data were analyzed using the 2^−ddCt^ method.

### shRNA transduction in fetal skeletal progenitor

The lentivirus was packaged using CXCL12 and Kitl specific shRNA constructs as previously described^40^ and following the protocol of JetPRIME (BIOPARC, France). The shRNA sequence was designed to against all three CXCL12 variants (Supplementary Table S3). The sorted fetal skeletal progenitors CD133−CD55− or CD133−CD55−Sca1− were re-suspended in MEM alpha medium with 15% FCS and transduced with the lentivirus carrying scramble control or shRNA constructs targeting CXCL12 or Kitl. Forty-eight hours after transduction, 1000–5000 GFP+ cells were sorted, mixed with 5 μl matrigel and then transplanted under KC. The grafts were harvested at one month for analysis. In addition, to detect gene knockdown efficiency, the transduced GFP+ cells were sorted into RNA lyses buffer for RNA isolation, cDNA synthesis and qPCR. Transduction was typically carried out with multiplicity of infection around five.

### Statistical Analysis

Statistical analyses were performed using GraphPad Prism software v.6. All data are presented as mean ± SD, and two group comparisons were done with a two-tailed Student’s t test. A value of p < 0.05 was taken as statistically significant.

## Additional Information

**How to cite this article**: Weng, L. *et al*. Identification of a CD133−CD55− population functions as a fetal common skeletal progenitor. *Sci. Rep.* **6**, 38632; doi: 10.1038/srep38632 (2016).

**Publisher's note:** Springer Nature remains neutral with regard to jurisdictional claims in published maps and institutional affiliations.

## Supplementary Material

Supplementary Information
