# Supplementary material for: Identification of a CD133−CD55− population functions as a fetal common skeletal progenitor
Source: Sci Rep. 2016 Dec 8;6:38632. doi: 10.1038/srep38632 (PMC5144148; doi:10.1038/srep38632)
Supplement: Supplementary Information [file srep38632-s1.pdf]

Identification of a CD133-CD55- population functions as a fetal common skeletal progenitor

Lihong Weng,[1,2,3,7] Xingbin Hu,[1,6,7] Bijender Kumar,[1,2] Mayra Garcia,[1,2] Ivan Todorov,[4] Xiaoman Jung,[1] Guido Marcucci,[1,2] Stephen J. Forman[2,3,5] and Ching-Cheng Chen[1,2,5,*]

[1]Divison of Hematopoietic Stem Cell and Leukemia Research, Gehr Family Center for Leukemia Research, Beckman Research Institute of City of Hope, Duarte, CA 91010, USA

[2]Department of Hematology and Hematopoietic Cell Transplantation, City of Hope, Duarte, CA 91010, USA

[3]Departments of Cancer Immunotherapeutic and Immunology,

[4]Department of Diabetes and Metabolic Diseases Research,

Beckman Research Institute of City of Hope, Duarte, CA 91010, USA

[5]Irell & Manella Graduate School of Biological Sciences, City of Hope, Duarte, CA 91010, USA

[6]Department of Transfusion Medicine, Xijing Hospital, Fourth Military Medical University, Xi'an 7100032, PR China.

[7]These authors contributed equally to this work

*Corresponding author: chingchengchen@coh.org (C.-C.C.)

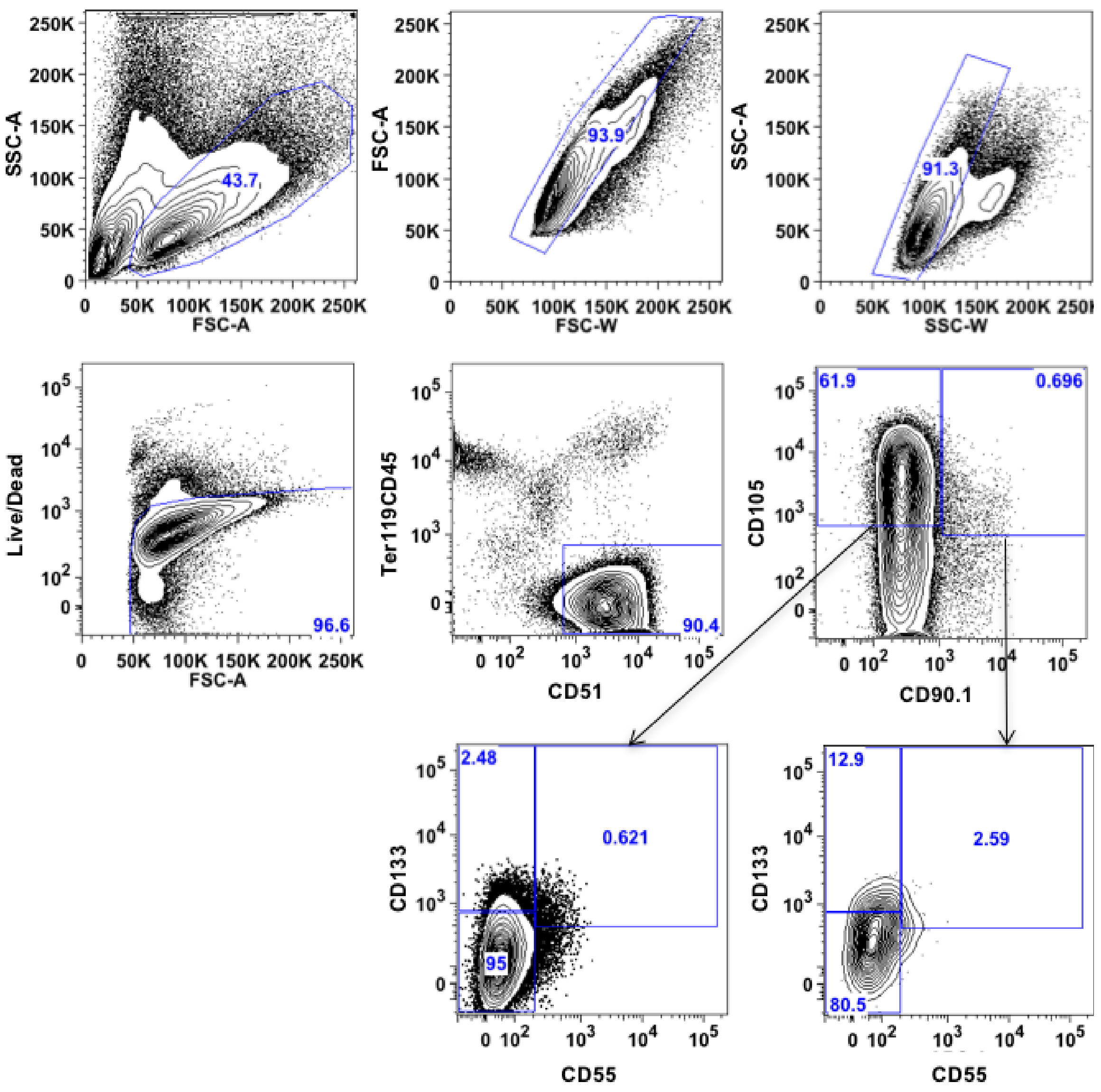

Supplementary Figure S1. The gating strategy for fetal skeletal progenitor analysis. The fetal limb was digested and stained with antibody cocktails for FACS analysis. The cells were selected first based on FSC and SSC value. Doublets and dead cells were gated out. The CD45+/Ter119+ positive cells were further excluded. CD105+CD90.1- osteochondral progenitor and CD105+CD90.1+ osteoprogenitors were gated from CD51+ cell population and then were further divided into three subpopulations, CD133-CD55-, CD133+CD55- and CD133+CD55+.

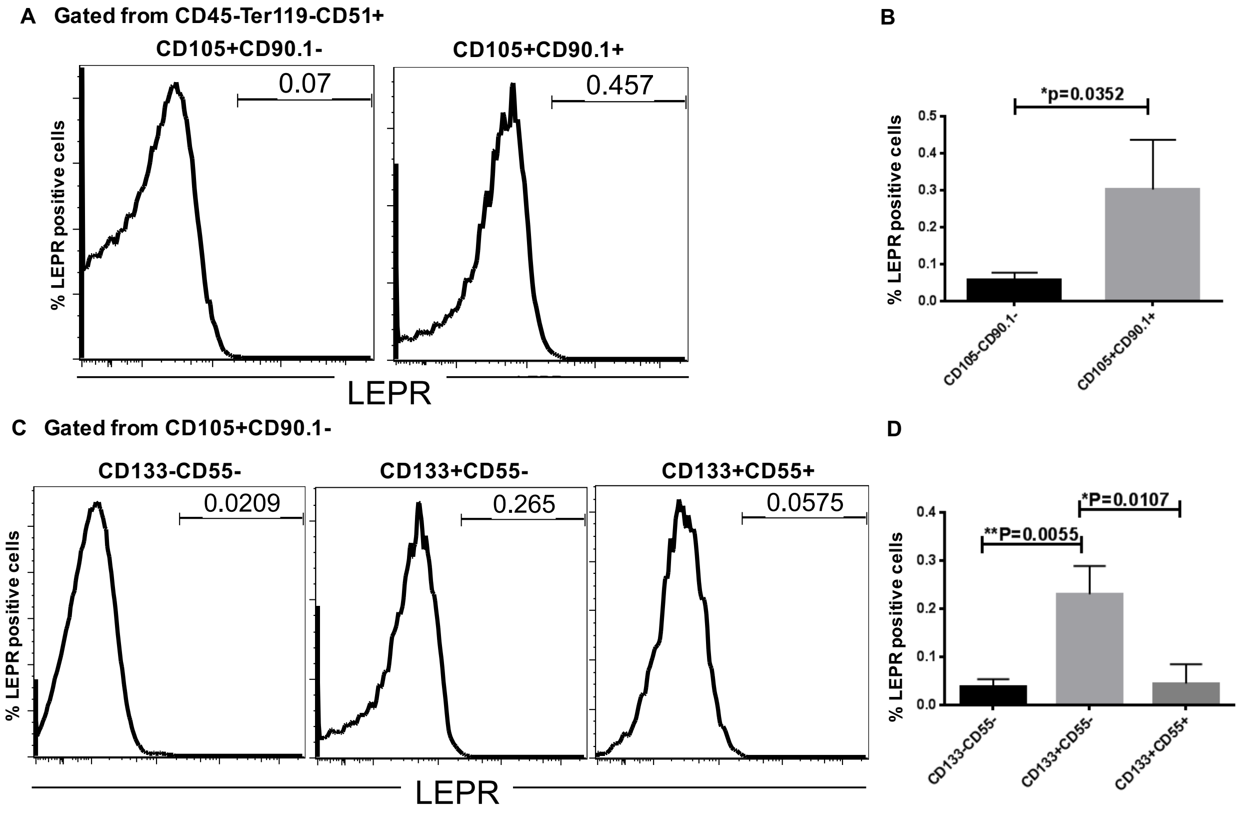

Supplementary Figure S2. Expression of LEPR in fetal skeletal progenitor. FACS analysis of LEPR expression in E14.5 osteochondral progenitors (CD105+CD90.1-) and osteoprogenitors (CD105+CD90.1). (**A**) Representative FACS analysis of LEPR expression in osteochondral and osteoprogenitors. (**B**) Distributions of LEPR positive cells in osteochondral and osteoprogenitors. (**C**) Representative FACS analysis of LEPR expression in three osteochondral progenitor derived subsets. (**D**) Distributions of LEPR positive cells in three subpopulations. (n=3 for each group. *p<0.05, **P<0.01.)

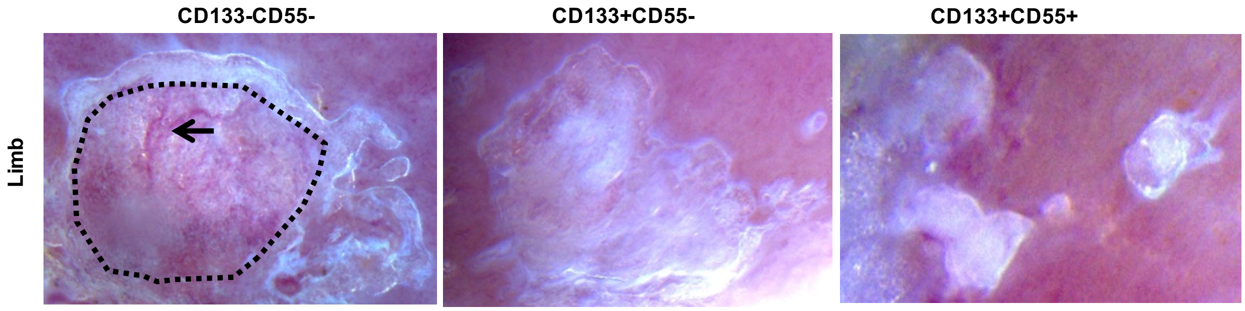

Supplementary Figure S3. Long term engraftments of fetal skeletal progenitors. E14.5 fetal CD133-CD55-, CD133+CD55- and CD133+CD55+ progenitors 4 months after transplanted under KC. Marrow formation were only identified in the graft derived from CD133-CD55- progenitor (dot line circled). Vasculatures (arrow) can be easily observed.

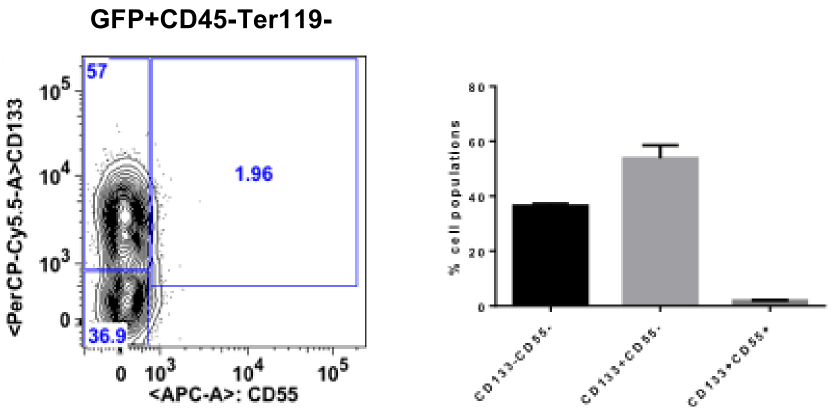

Supplementary Figure S4. CD133-CD55- fetal progenitor gave rise to CD133+CD55- and CD133+CD55+ cell *in vivo*. Representative FACS profiles of CD133-CD55- fetal progenitor cell derived KC graft one month after transplantation (n=3 for transplantation experiments).

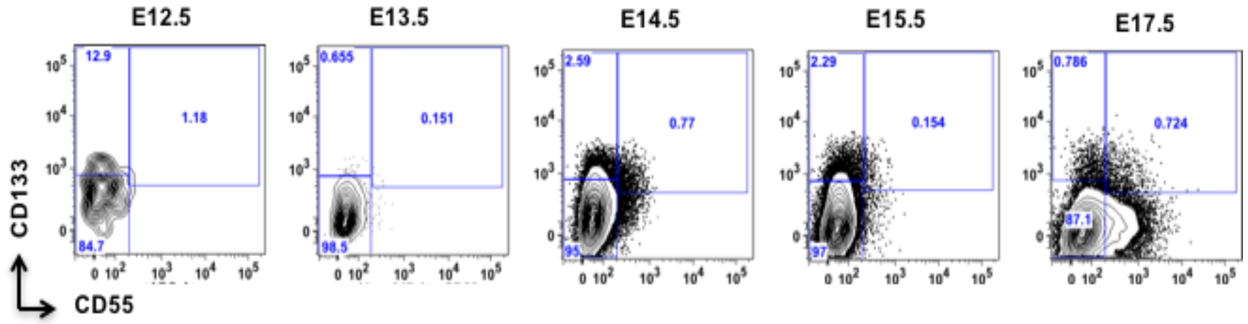

Supplementary Figure S5. CD133 and CD55 expression on fetal skeletal cells are tightly regulated during development. Representative FACS analyses of fetal limb cells at indicated time point.

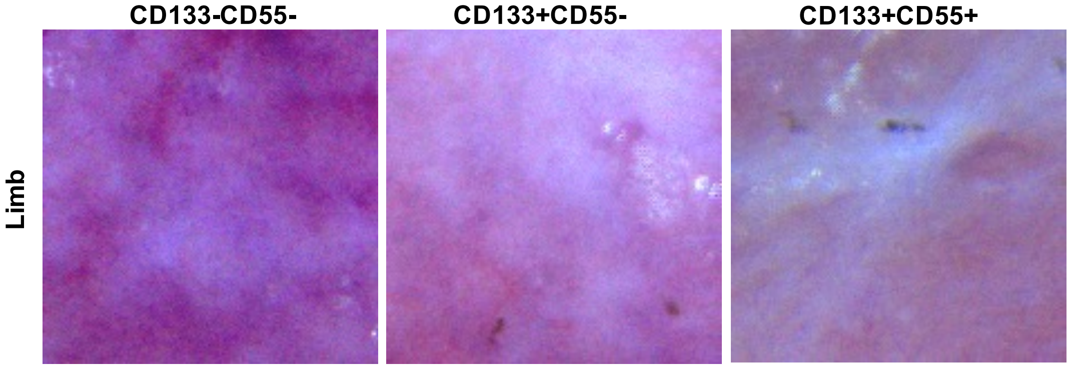

Supplementary Figure S6. E12.5 fetal limb cells can not initiate ectopic HSC niche. Sorted CD133-CD55-, CD133+CD55- and CD133+CD55+ cells from E12.5 limbs were transplanted under KC and harvested after 1 month. No donor contributions were detected.

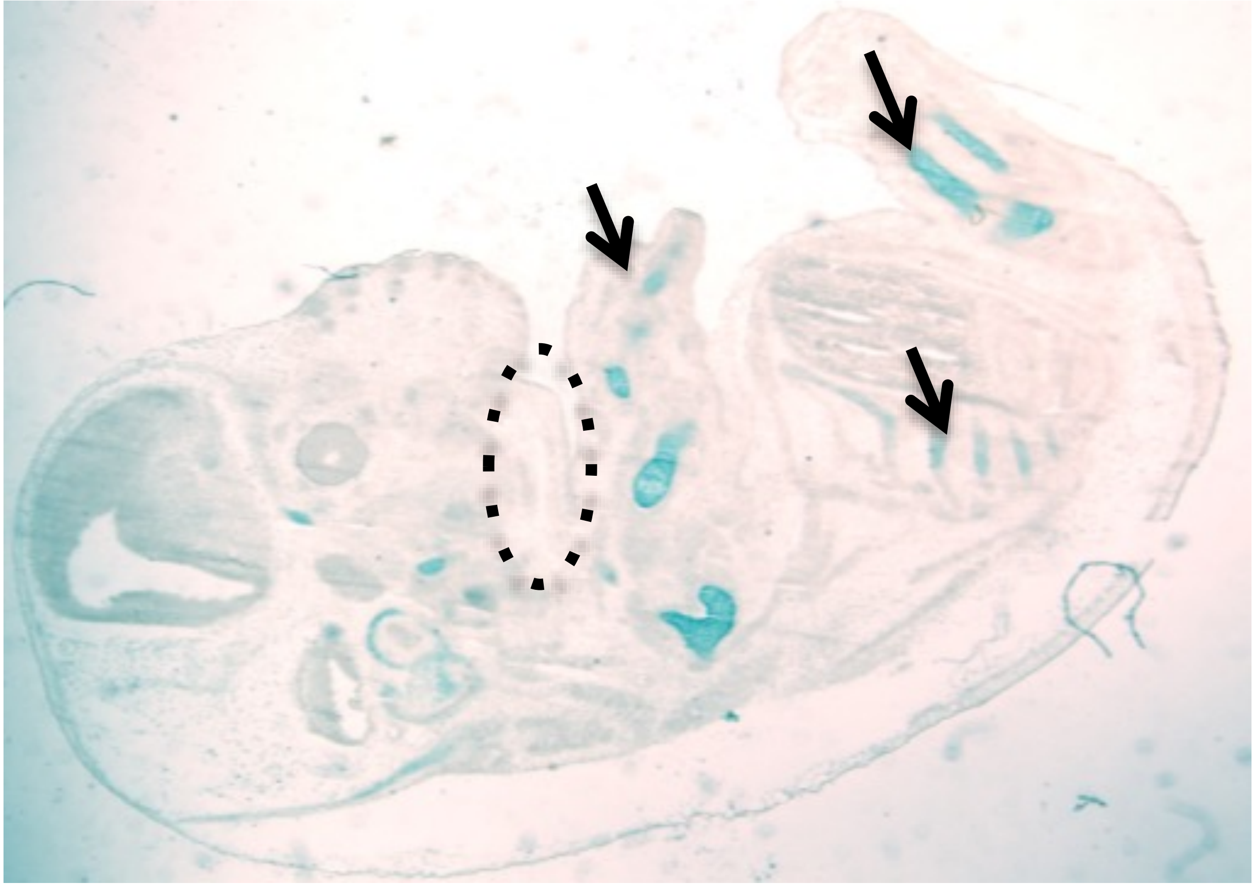

Supplementary Figure S7. Distribution of chondrocytes in E14.5 fetus. Representative whole mount section of E14.5 fetus was stained with pentachrome (yellow=osteoid, blue= cartilage) staining. Only chondrocyte clusters (Arrows) were observed in fetal limbs and ribs (blue regions). No chodrogensis were observed in mandible (dot-line circle).

	CD133-CD55-	CD133+CD55-	CD133+CD55+
Bone formation	12/13	3/3	3/3
Marrow formation	12/13	0/3	0/3
Adipocytes (GFP)	0/4		

Supplementary Table S1. Summary of kidney capsule transplantation results. The proportion of transplants with bone, marrow or adipocyte formation is given as (number of transplants with positive phenotype) / (total number of transplantation performed).

Primers	Sequence
CD133	5'- CTCATGCTTGAGAGATCAGGC-3′ For
	5'-CGTTGAGGAAGATGTGCACC-3′ Rev
CD55	5'-ACTGTTGATTGGGACGATGAG -3′ For
	5'-TGGTGGCTCTGGACAATGTA-3’ Rev
GAPDH	5′-GGAAGGTGAAGGTCGGAGTC-3′ For
	5′-TTCCCGTTCTCAGC-CTTGAC-3′ Rev

Supplementary Table S2. The PCR primers used in this study.

shRNA	Sequences
Kitl	5'- tGAAACAGTCAAGTCTTACAttcaagagaTGTAAGACTTGACTGTTTCtttttc -3' For
	5'- tcgagaaaaaaGAAACAGTCAAGTCTTACAtctcttgaaTGTAAGACTTGACTGTTTCa -3' Rev
CXCL12	5'- tGTGCATTGACCCGAAATTAttcaagagaTAATTTCGGGTCAATGCACtttttc -3' For
	5' – tcgagaaaaaaGTGCATTGACCCGAAATTAtctcttgaaTAATTTCGGGTCAATGCACa -3' Rev
Scramble sequence	5'- tGAGTTAGGCGTAGTCGAATttcaagagaATTCGACTACGCCTAACTCtttttc -3' For
	5'- tcgagaaaaaaGAGGTTAGGCGTAGTCGAATtctcttgaaATTCGACTACGCCTAACTCa -3' Rev

Supplementary Table S3. The Oligos used to generate shRNA constructs targeting Kitl and CXCL12.

The 19 bp gene target specific sequences are indicated in capital.
